# The association between multilingualism and cognitive function among literate and illiterate older adults with low education in India

**DOI:** 10.1002/bsa3.70018

**Published:** 2025-05-15

**Authors:** Iris M. Strangmann, Emily Briceño, Sarah Petrosyan, Erik Meijer, Emma Nichols, Lindsay C. Kobayashi, Shrikanth Narayanan, Jinkook Lee, Miguel Arce Rentería

**Affiliations:** 1 Taub Institute for Research on Alzheimer’s Disease and the Aging Brain, Department of Neurology, Columbia University College of Physicians and Surgeons, New York City, New York, USA; 2 Department of Physical Medicine & Rehabilitation, University of Michigan Medical School, Ann Arbor, Michigan, USA; 3 Center for Economic and Social Research, University of Southern California, Los Angeles, California, USA; 4 Leonard Davis School of Gerontology, University of Southern California, Los Angeles, California, USA; 5 Department of Epidemiology, University of Michigan School of Public Health, Ann Arbor, Michigan, USA; 6 Survey Research Center, Institute for Social Research, University of Michigan, Ann Arbor, Michigan, USA; 7 Signal Analysis and Interpretation Laboratory, University of Southern California, Los Angeles, California, USA; 8 Department of Economics, University of Southern California, Los Angeles, California, USA

**Keywords:** aging, bilingualism, cognition, education, illiteracy, literacy, multilingualism

## Abstract

**Introduction:**

This study examined whether multilingualism modified the adverse effect of illiteracy on cognition among older Indian adults with low to no formal schooling.

**Methods:**

Data came from the Longitudinal Aging Study in India-Diagnostic Assessment of Dementia (*N* = 2533, *M*age = 70.3 years, 84% illiterate, 24% multilingual, ≤4 years of education). Generalized linear models and propensity score matching assessed whether multilingualism modified the association between illiteracy and domain-specific cognition.

**Results:**

Illiteracy was negatively associated with cognition, while multilingualism was positively associated. While multilingualism did not buffer the association of illiteracy, it did increase the positive association of literacy with executive functioning and memory.

**Discussion:**

These findings highlight the detrimental effects of illiteracy on cognition, while multilingualism appears beneficial among older adults with low to no formal schooling. The benefits of multilingualism, however, were diminished in illiterate compared to literate older adults, suggesting that limited early-life educational opportunities can weaken its contribution to cognition.

## 1 │ BACKGROUND

Bilingualism and/or multilingualism might protect against cognitive aging due to the additional language control required to resolve cross-linguistic conflict.^1^ The existing evidence for this claim, however, is currently inconclusive. Several meta-analyses revealed no systematic support for the relationship between cognition and bi- or multilingualism (henceforth multilingualism) when controlling for publication bias or other confounding factors.^2^ Moreover, since multilingualism is not randomly assigned throughout the life course, it is difficult to isolate its contribution to cognition, independently of other often covarying factors, such as educational opportunities, biculturalism, migration experiences, and SES.^3^

Much of the existing evidence on multilingualism and cognitive aging comes from populations in Western Europe and North America.^4^ Much less is known about multilingualism and cognition among people from low- and middle-income countries (LMICs), despite the fact that LMICs are currently undergoing the greatest increase in population aging.^5^ LMICs also have larger disparities in healthy life expectancy than high-income countries,^6^ and two-thirds of the world’s older adults with Alzheimer’s disease and related dementias (ADRD) are projected to reside in LMICs by 2050.^7^

Research from LMICs may provide unique insights into the role of multilingualism in cognitive aging due to rich linguistic diversity and an inherent societal presence of multilingualism.^8^ In India, for example, around 26% of the population is estimated to be multilingual, although actual multilingual rates may be higher due to differences in defining language versus dialect.^9^ Moreover, India has significant linguistic diversity, reporting 122 different languages as major languages (spoken by more than 10,000 people), in addition to 1599 other languages.^10^ This diversity is further exemplified by Indian languages coming from a range of different language families, including Indo-Aryan, Dravidian, Austro-Asiatic, Tibeto-Burmese, and Andamanese.^8^ Furthermore, the sociodemographic and linguistic diversity within India offers opportunities to study multilingualism among individuals with limited formal education and literacy skills, providing insight into the contribution of multilingualism to cognition among underrepresented populations while reducing the effects of educational and socioeconomic factors confounding the associations of multilingualism with cognition.

Over the past 70 years, India has more than quadrupled its national literacy rate, increasing from 18% in 1951^11^ to 78% in 2022.^12^ Among the older population, however, illiteracy rates remain high: Approximately 63% of older adults in India are illiterate, but this projection increases to 95% among older adults who lack formal schooling.^13^ Prior studies found that illiteracy alone increased dementia risk, even when compared with literate adults with limited education.^14^ Given that illiteracy disproportionately burdens the older segment of the population within India, placing them at additional risk for detrimental late-life cognitive outcomes, it is critical to determine factors that might provide cognitive resilience within this at-risk population. India, thus, provides a unique opportunity to examine the extent to which multilingualism has the potential to buffer the detrimental effect of illiteracy on cognition.

In this study, we evaluated the independent and synergistic contributions of multilingualism and illiteracy to cognitive functioning among older Indian adults with little to no formal schooling. Multilingualism is defined broadly as when individuals use two or more languages in their everyday lives.^15^ To distinguish between the effects of illiteracy and those of little to no formal education, we evaluated illiterate and literate multilingual and monolingual older adults that had no more than 4 years of formal schooling. We chose this lower educational attainment cut-off given that it is the average years of education of the older adults in our study’s cohort (*M* = 3.8), represents around a quarter of the literate adults within the cohort, and has been used in prior studies of illiteracy and cognitive decline.^16^ We hypothesized that, independently of the years of education and relevant confounders, multilingualism would be associated with better cognitive performance, and illiteracy would be associated with worse cognitive performance. We also hypothesized that multilingualism would modify the association of illiteracy with cognition, such that associations between illiteracy and cognition would be weaker among multilingual participants compared to monolingual participants.

## 2 │ METHODS

### 2.1 │ Participants

Participants were from the Longitudinal Aging Study in India-Diagnostic Assessment of Dementia (LASI-DAD). LASI-DAD is a subsample of adults aged 60 and older from the Longitudinal Aging Study in India (LASI), a nationally representative study examining aging within India. LASI-DAD includes community-residing older adults from 18 states and union territories in India (*n* = 4096). Testing is available across 12 languages: Hindi, Kannada, Malayalam, Gujarati, Tamil, Punjabi, Urdu, Bengali, Assamese, Odiya, Marathi, and Telugu. The study visit was carried out in the participants’ preferred language within the available 12. To facilitate accurate assessment due to the linguistic diversity inherent in India, all LASI-DAD examiners were recruited from the states in which testing was conducted, such that they were multilingual in the local languages. LASI-DAD oversamples individuals at high risk of cognitive impairment to ensure greater numbers of adults with dementia and mild cognitive impairment in the sample. See Lee and colleagues for additional details regarding the LASI-DAD sampling procedure.^16^

The current study included participants with 4 or fewer years of education who completed a comprehensive cognitive assessment and self-reported literacy and multilingualism status (*n* = 2553, 84% illiterate, 24% multilingual). We included only participants with less than/equal to the average years of education within LASI-DAD (*M* = 3.8 years) to reduce the confounding influence of years of education on cognition from that of acquiring literacy. Analyses were conducted using the harmonized LASI-DAD Wave 1 data (version A.3) available on request from the Gateway to Global Aging Data website (https://g2aging.org/).

### 2.2 │ Measures

#### 2.2.1 │ Multilingualism

Participants self-reported their mother tongue (a common term within the Indian census denoting an individual’s first language) and any additional spoken language(s). We classified participants as monolingual if they reported only one language and as multilingual if they reported more than one language.

#### 2.2.2 │ Illiteracy

Participants were asked to report if they were able to read and write. We classified participants as illiterate if they answered “no” and literate if they answered “yes.”

#### 2.2.3 │ Cognition

LASI-DAD administers a comprehensive neuropsychological battery based on the Harmonized Cognitive Assessment Protocol (HCAP) adapted to be linguistically and culturally appropriate for India.^17^ The battery evaluates the cognitive domains of executive functioning, language, memory, orientation, and visuospatial abilities. Table 1 shows the assessments used within the LASI-DAD battery by cognitive domain.

Several tests were adapted to account for literacy so as to not place illiterate participants at a disadvantage. Within the language domain, literate participants were asked to write a complete sentence and follow a read command, whereas illiterate participants were asked to say a full sentence (*please tell me something about your house*) and imitate an examiner’s behavior (i.e., closing eyes for 3 s). Domain-specific cognitive factor scores were derived using a confirmatory factor analysis. The resulting standardized scores have a mean of 0 and standard deviation (SD) of 1. Previous work supported the cognitive factor structure of the battery and demonstrated comparable measurement across the study languages. See Gross and colleagues for additional details on deriving the cognitive factor structure.^18^

#### 2.2.4 │ Sociodemographic covariates

Participants’ age was derived using the LASI-DAD interview month and year and the participants’ birth month and year. Years of education were self-reported. We used parental years of education as a proxy for childhood socioeconomic status (SES). The education of the mother and father was self-reported by participants using categories varying from *never attended school* to *post-graduate degree*. The participants’ residency at the time of the interview was coded as urban or rural following its classification in the 2011 Indian census data. Current SES was further characterized using data on participants’ consumption. Consumption is a measure of the use of goods and services by households divided into quartiles, calculated using the total household consumption, which includes food consumption in the last week, non-food consumption in the last 30 days, other non-food consumption in the past year, outpatient health expenditures in the past 30 days, and inpatients’ healthcare expenditures in the past year. Taken together, consumption is a better proxy for SES, as it more readily reflects a household’s standard of living, while income data may be subject to greater variability.^19^

## 3 │ ANALYSES

Descriptive statistics were calculated across the four groups: monolingual-illiterate, multilingual-illiterate, monolingual-literate, and multilingual-literate participants. One-way ANOVA and chi-squared tests evaluated differences between groups in covariates. The associations between multilingualism and illiteracy and cognitive domain scores were evaluated using two approaches. First, separate linear regressions evaluated the association of multilingualism (monolingual [reference] vs multilingual), illiteracy (literate [reference] vs illiterate) and their interaction for each of the cognitive domains of executive functioning, memory, language, and visuospatial abilities. Second, we added a propensity score matched analysis as an additional approach to account for confounding between mono- and multilingual groups, beyond covariate model adjustments. Since some variables often covary with multilingualism, these factors may not be independent of the grouping variable, in which case propensity score matching is a more appropriate approach to reduce the possibility of spurious effects.^20^ The linear regression models were repeated on the matched sample.

Propensity scores were estimated based on literacy, age, sex/gender, years of education, parental education (mother and father), consumption, and rurality. The propensity scores were estimated by matching the multilingual to the monolingual group, using logistic regression, applying one-to-one nearest-neighbor matching without replacement. All multilingual participants (*n* = 605) were matched to a monolingual participant, so that the total matched sample comprised 1210 older adults of which 50% were monolingual and 50% were multilingual. A good balance was achieved, with all standardized mean differences below 0.1 after matching. No statistically significant differences remained between multilingual and monolingual groups after matching in any of the covariates, yet we retained adjustments in all subsequent linear regressions for participants’ age, sex/gender, years of education, parental education, consumption, and rurality. The matched sample’s demographic characteristics are provided in Table S1. All analyses were conducted in R version 4.3.1.

## 4 │ RESULTS

On average, participants were 70.3 years old (SD = 8.0) and 65% female, with 71% residing in rural areas. The average years of education was 0.65 years (SD = 1.3), with 50% of participants having 1 year or less of formal education. Prior to matching, across multi- and monolingual participants, the literate group had more years of education, fewer female participants, and higher childhood SES than the illiterate group. Multilingual-literate participants were less likely to reside in rural areas than all other groups. The groups did not differ in age and current SES. The sample’s demographic characteristics are provided in Table 2.

### 4.1 │ Multilingualism, literacy, and cognition

**FIGURE 1:**
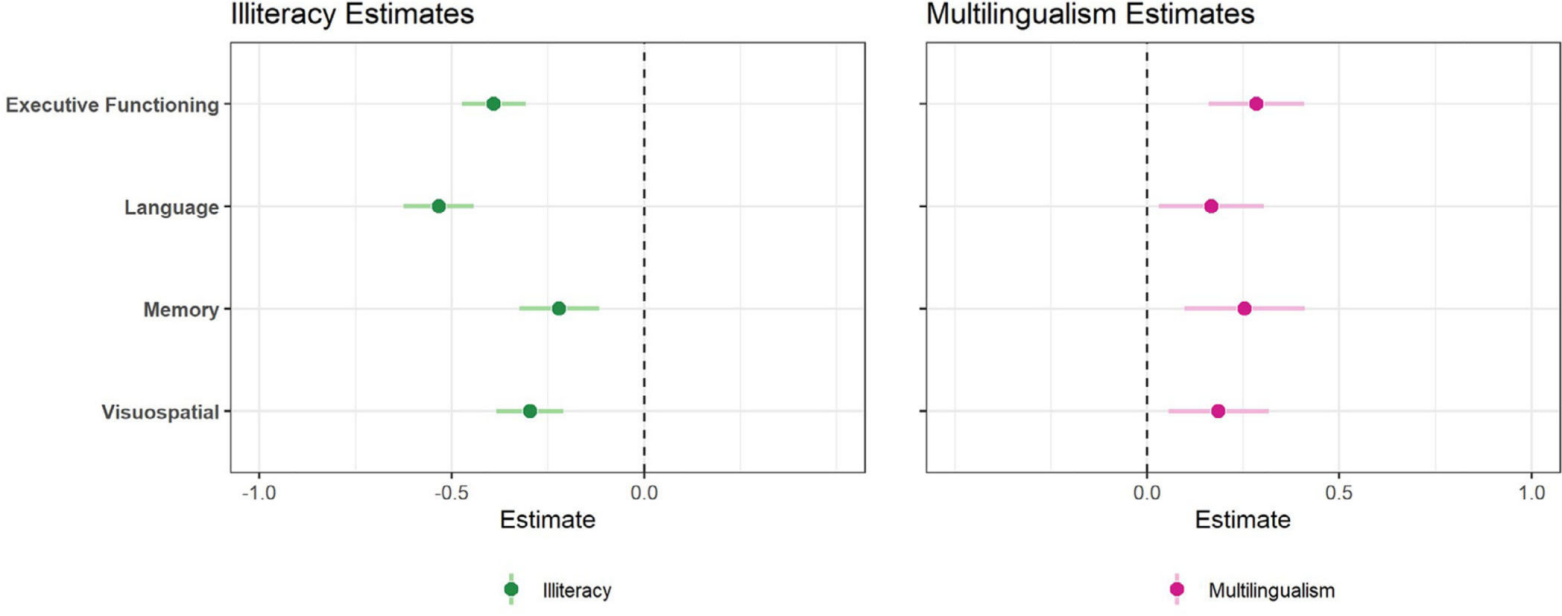
Full sample adjusted main effects estimates and 95% confidence intervals for illiteracy and multilingualism per cognitive domain. Reference: literate, monolingual.

**FIGURE 2:**
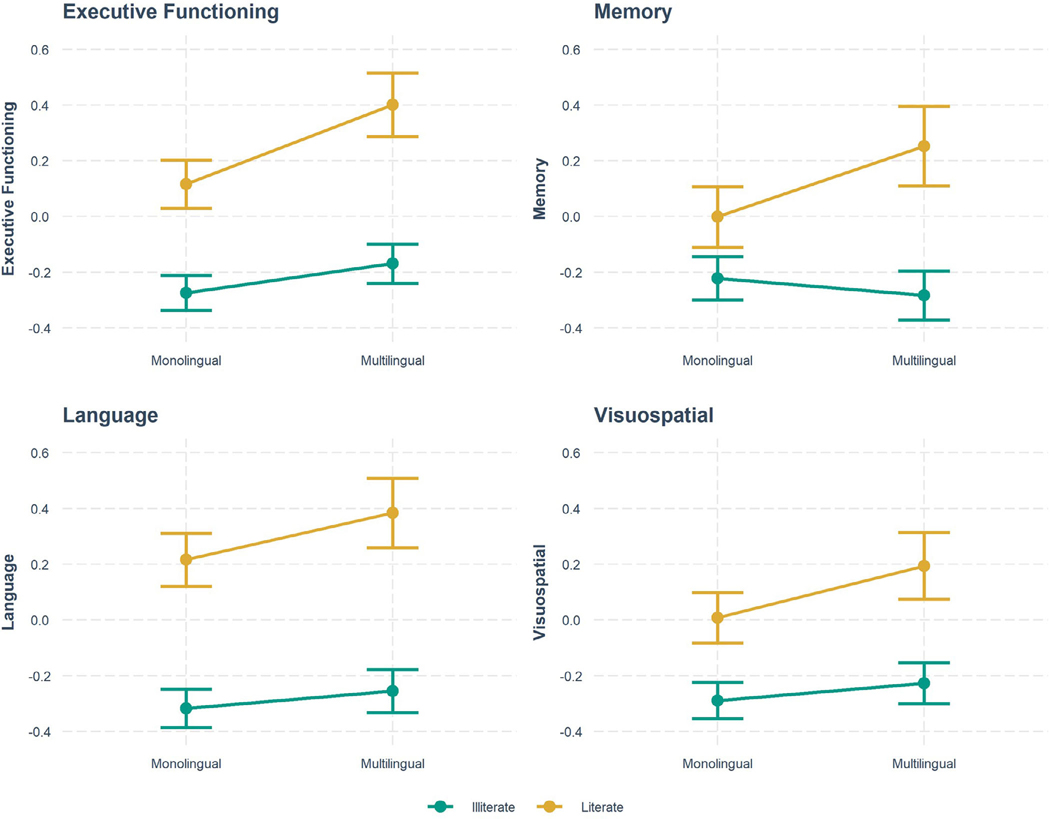
Associations of illiteracy and multilingualism across cognitive domains (estimates and 95% confidence intervals).

Within the full sample, multilingual participants outperformed monolingual participants in all cognitive domains: executive function (*B* = 0.28 [0.16, 0.41]), language (*B* = 0.17 [0.03, 0.30]), memory (*B* = 0.25 [0.10, 0.41]), and visuospatial abilities (*B* = 0.19 [0.06, 0.32]; Table 3, Figure 1). Illiterate participants performed worse than literate participants in all cognitive domains: executive function (*B* = −0.39 [−0.47, −0.31]), language (*B* = −0.53 [−0.62, −0.44]), memory (*B* = −0.22 [−0.32, −0.12]), and visuospatial abilities (*B* = −0.30 [−0.38, −0.21]; Table 3, Figure 1). There was a significant multilingualism and illiteracy interaction in executive function (*B* = −0.18 [−0.32, −0.04]) and memory (*B* = −0.14 [−0.19, −0.09]), but not within language or visuospatial abilities (*p* > .05), although effect estimates were in a consistent direction (Table 3, Figure 2).

To evaluate the interactions, we examined the estimates of executive functioning and memory performance across the four groups (monolingual-illiterate, multilingual-illiterate, monolingual-literate, and multilingual-literate participants) after covariate adjustments. All groups differed in executive functioning performance. The monolingual-illiterate group (reference) performed worst on executive functioning, followed by the multilingual-illiterate group (*B* = 0.11 [0.05, 0.17]), the monolingual-literate group (*B* = 0.39 [0.31, 0.47]), and the multilingual-literate group (*B* = 0.68 [0.56, 0.79]). For memory, however, the multilingual-literate participants (*B* = 0.48 [0.33, 0.62]) outperformed all other groups, followed by the monolingual-literate participants (*B* = 0.22 [0.12, 0.32]), while neither illiterate monolingual nor illiterate multilingual participants differed from each other (*B* = −0.06 [−0.14, 0.01]). Tukey HSD post hoc tests revealed that multilingual-literate participants outperformed monolingual-literate participants in both executive functioning (*B* = 0.29 [0.12, 0.45]) and memory (*B* = 0.25 [0.05, 0.46]).

The results of the propensity score matched analysis aligned with the findings ofthe full sample analysis. Illiterate participants performed worse than literate participants in all cognitive domains (all *p* < .05), and multilingual participants outperformed monolingual participants in all cognitive domains. Again, we observed an interaction between multilingualism and illiteracy within memory and executive functioning. Model estimates for main effects, interactions, and covariates per cognitive domain of the propensity score matched analysis are provided in Table S2.

## 5 │ DISCUSSION

Among older adults with little to no formal schooling in India, multilingualism was cross-sectionally associated with better cognitive performance, while illiteracy was associated with worse cognitive performance across multiple cognitive domains. The hypothesis that multilingualism might buffer the negative impact of illiteracy on cognition, however, was not supported. Although we did observe an interaction between multilingualism and illiteracy on executive functioning and memory, results indicated that this was mostly driven by multilingual *literate* older adults outperforming all other groups. In addition, multilingual *illiterate* older adults did not differ on memory performance and demonstrated only slightly better executive functioning compared to their monolingual counterparts. Therefore, our results suggest that while multilingualism might be beneficial to cognition among literate older adults with limited schooling, its potential cognitive benefit is either weaker or absent among illiterate older adults.

Previous studies have observed better cognitive performance among multilingual relative to monolingual older adults across wide education gradients, albeit typically higher than our sample.^21–23^ However, this evidence is not consistent, as illustrated by existing meta-analyses.^2^ Arguments against a multilingual cognitive benefit have largely focused on unequal distribution of relevant socioeconomic confounds between multilingual and monolingual adults, such as years of education, migration experiences, and childhood SES.^24^ Unlike most studies examining multilingualism, which were largely conducted in high-income countries,^25^ our study leveraged a large community-dwelling sample of educationally disadvantaged adults residing in India. Our multilingual and monolingual groups were well characterized in terms of sociodemographic and socioeconomic factors, and – given that we selected for participants with low to no formal schooling – our groups were fairly comparable across these factors. In addition, through propensity score matching, we were able to more conservatively account for differences in confounds, and our results remained unchanged. Similarly, we posit that these findings are not the result of a healthy migrant effect^26^ since multilingualism in our sample was not driven by immigration (i.e., less than 2% of the LASI-DAD sample is born outside of India) but, rather, is a consequence of multilingualism being deeply ingrained within Indian society and, therefore, occurring at all sociodemographic levels.

That said, illiterate older adults benefited less from multilingualism than literate older adults, particularly in the domains of executive functioning and memory. The limited research among multilingual illiterate individuals and those with limited educational attainment generally suggests better late-life cognitive outcomes for multilingual people compared to their monolingual counterparts. For instance, Alladi and colleagues found a delay in dementia onset among multilingual illiterate individuals compared to their monolingual illiterate adults in India.^27^ Similarly, Gollan and colleagues observed a later dementia onset among multilingual individuals with lower years of schooling (i.e., below 6 years of education) compared to multilingual individuals with higher education levels in the United States.^28^ However, a recent study by our group found the opposite pattern, with multilingual older adults who had higher levels of education experiencing greater cognitive benefits compared to their peers with lower levels of education.^29^

In our sample, multilingualism was associated with better language and visuospatial abilities across both literate and illiterate adults, but this association was weaker and did not reach statistical significance in executive functioning and memory. For illiterate individuals, multilingualism only provided a small benefit in executive functioning, while offering no benefit in memory. There may be a few explanations for these differential effects. First, the negative effect of illiteracy on cognition was much larger than the positive effect of multilingualism. This may indicate that a threshold to the cognitive contribution of multilingualism could not be reached in the face of limited early-life educational opportunities, such as illiteracy, which may diminish the cognitive returns of multilingualism. Indeed, some have suggested that multilingual cognitive benefits compete with other cognitive exposures that apply to both mono- and multilingual people.^30^ Considering the influence of both illiteracy and multilingualism, the detrimental effects of illiteracy have been reliably shown across populations globally,^31,32^ while studies greatly conflict on the presence of multilingual benefits and report these to be small, if present.^3^ Under this competing-exposures account, it is possible that the detrimental effects of illiteracy cancel out a potential, small benefit of multilingualism. Second, there may be differential effects of multilingualism related to demonstrating proficiency across all modalities (i.e., reading, writing, speaking, and understanding) versus just in informal modalities (i.e., speaking, understanding). It is unclear whether the multilingual literate adults in our sample were literate in their other languages as well and whether their proficiency in speaking differed from that of multilingual illiterate adults. Between-group differences in aspects of multilingualism such as proficiency might explain differences between multilingual literate and illiterate adults.

The adverse effects of illiteracy on cognitive performance were large and extended to all cognitive domains, which align with findings from previous studies of illiterate adults outside of India.^14,31,32^ Importantly, our study observed the detrimental effects of illiteracy, by and large independently of the confounding influence of years of education, which was on average less than a year across all participants. Since literacy and education are naturally entwined, it is difficult to tease apart the contribution of acquisition of literacy skills on cognitive functioning from that obtained through varied educational experiences. However, our findings indicate that, independently of education, illiteracy is associated with worse cognitive functioning in late life.

There are several – non-mutually exclusive – explanations as to why illiteracy may be associated with worse cognitive functioning. First, illiteracy can restrict an individual’s ability to engage in cognitively stimulating leisure activities and occupational opportunities and obtain higher socioeconomic status, which have all been associated with better late-life cognitive outcomes.^26,33–35^ Under this explanation, the adverse effects of illiteracy would be a consequence of sociodemographic disadvantages, resulting in worse cognitive health, placing illiterate individuals at greater risk of cognitive decline than literate individuals. Future studies should investigate the role of sociocultural factors such as leisure activities, occupational history, and SES.

In addition to sociodemographic mechanisms, the ability to read and write has also been linked to neurobiological changes. For example, illiteracy has been associated with lower fractional anisotropy values, which may reflect reduced white matter microstructure integrity.^36^ Furthermore, improving literacy skills in illiterate individuals has led to increases in gray matter volumes and white matter integrity, suggesting structural changes following literacy training in adulthood.^37^ Future studies should evaluate whether the adverse cognitive effects of illiteracy are the result of differences in cortical structures and function.

Important strengths of our study are that the sample is based on a nationally representative cohort and that it consists of underrepresented individuals, who are highly linguistically diverse, including understudied languages (e.g., Urdu, Telugu, Kannada). However, limitations of the present study should also be considered. We treated multilingual adults as a monolithic group, despite the heterogeneity within multilingual populations.^38^ Our estimates, therefore, reflect “average” multilingualism effects across these linguistic dimensions. Assessing multilingual individuals’ language background – such as age of acquisition and frequency of language use – may help elucidate which aspects of multilingualism are associated with better cognitive aging. In low-literacy populations, this extends to the modality of language use (oral vs written) and whether literate abilities extend to one, some, or all languages spoken. We recommend that future studies examine these within-group multilingual differences in linguistic characteristics and their role in cognition, particularly among linguistically diverse populations such as those found in India. Moreover, illiteracy is assessed as a binary variable (literate vs illiterate), whereas in reality, literate abilities exist on a continuum.

Although this approach does not fully capture the spectrum of literacy abilities, our self-reported illiteracy rates align closely with national estimates (e.g., 95% illiteracy among older adults without formal schooling according to the National Sample Survey 2008 vs 94% in our sample) and is associated with significant decrease in years of education, increase in the presence of females, and increase in rurality, which are described causes of illiteracy in India,^12^ suggesting that our classification reflects literacy trends in India among this population. Additionally, we capped years of education at 4 for both literate and illiterate individuals, reducing the likelihood of including participants with higher literacy levels in the literate group. Future research, including analyses using objective literacy assessments from Wave 2 of LASI-DAD, will help refine the measurement of literate abilities as a continuous construct and provide deeper insights into its role in cognitive aging. Lastly, assessing cognition in illiterate individuals is challenging when many neuropsychological assessments require (some degree of) literacy.^39^ Due to this concern, we adapted our assessments to be appropriate and valid for illiterate individuals, such that they would not be placed at a disadvantage reflecting their inability to read or write during cognitive testing. Differences in measures between literate and illiterate participants were then incorporated into the estimation of domain factor scores, which are a more robust indication of cognitive performance than a single measure.^40^ The construct validity of our cognitive battery was previously established among our linguistically and educationally diverse cohort.^17^ Similarly, prior studies have established the validity of similar neuropsychological batteries to detect cognitive change among illiterate individuals and those with diverse educational backgrounds.^14^

## 6 │ CONCLUSION

Within the linguistically diverse and characteristically multilingual context of India, among an underrepresented sample of older adults with little to no formal schooling, multilingualism was associated with better late-life cognitive functioning. However, multilingualism might not be able to provide a cognitive benefit in the face of the detrimental impact of illiteracy. These findings warrant further investigation of the association between multilingualism and cognitive aging, especially among linguistically and educationally diverse populations.

## Supplementary Material

Supplementary Tables

Additional supporting information can be found online in the Supporting Information section at the end of this article.

## Figures and Tables

**TABLE 1:** LASI-DAD cognitive test assessments by domain factor

Domain	Assessment
Orientation	Orientation to time and place (e.g., day, month, year, city, state)
Memory	10-word recall, immediate, delayed, and recognition
	Three-word recall, immediate and delayed
	Brave Man, immediate and delayed recall
	Logical memory, immediate and delayed recall, and recognition
	Constructional praxis, delayed recall
Executive functioning	Raven progressive matrices
	Go/no-go trials 1 and 2
	Serial 7s
	Backward day naming
	Symbol cancelation
	Digit span, backward and forward score
Language	Naming common objects
	Animal naming
	Writing or saying a sentence
	Reading or repeating a phrase
	Close your eyes
	Paper-folding three-stage task
	Naming described objects
Visuospatial	Interlocking pentagons
	Constructional praxis, immediate
	Clock drawing

**TABLE 2:** Demographic characteristics across language and literacy status (*n* = 2553).

Predictor	Monolingual-Illiterate *n* = 1653	Multilingual-Illiterate *n* = 484	Monolingual-Literate *n* = 295	Multilingual-Literate *n* = 121
Age, *M* (SD)	70.4 (8.1)	69.7 (7.8)	70.9 (8.4)	70.6 (7.4)
Sex/gender, percentage female	71%	67%	47%	33%
Years of education	0.3 (0.9)	0.4 (1.0)	2.3 (1.7)	2.3 (1.6)
Parental education				
*Mother*	98% no school	98% no school	92% no school	95% no school
*Father*	89% no school	85% no school	75% no school	72% no school
Rurality, percentage rural	77%	62%	62%	49%
Consumption median (interquartile range)	2 (2)	2 (2)	2 (2)	2 (2)

**TABLE 3:** Adjusted model estimates per domain of full sample.

Predictor	Executive function		Language		Memory		Visuospatial	
	B	CI	B	CI	B	CI	B	CI
Illiteracy	−0.39***	−0.47, −0.31	−0.53***	−0.62, −0.44	−0.22***	−0.32, −0.12	−0.30***	−0.38, −0.21
Multilingualism	0.28***	0.16, 0.41	0.17*	0.03, 0.30	0.25**	0.10, 0.41	0.19**	0.06, 0.32
Illiteracy * Multilingualism	−0.18*	−0.32, −0.04	−0.11	−0.26, 0.04	−0.32***	−0.49, −0.14	−0.12	−0.27, 0.02
*Sex/gender*	−0.24***	−0.29, −0.19	−0.09**	−0.14, −0.03	0.01	−0.05, 0.07	−0.14***	−0.19, −0.09
*Rurality*	−0.17***	−0.22, −0.12	−0.11***	−0.17, −0.06	−0.21***	−0.28, −0.15	−0.02	−0.07, 0.04
*Age*	−0.02***	−0.02, −0.02	−0.02***	−0.02, −0.01	−0.03***	−0.03, −0.02	−0.02***	−0.02, −0.01
*Years of education*	0.09***	0.07, 0.11	0.06***	0.04, 0.08	0.07***	0.04, 0.09	0.06***	0.04, 0.08
*Mother education*	−0.05	−0.13, 0.04	−0.05	−0.15, 0.04	−0.03	−0.14, 0.08	−0.05	−0.14, 0.05
*Father education*	0.05***	0.02, 0.08	0.03	0.00, 0.06	0.05*	0.01, 0.09	0.06***	0.03, 0.09
*Consumption*	0.03**	0.01, 0.05	0.02*	0.00, 0.05	0.06***	0.04, 0.09	0.02	0.00, 0.04
Adjusted *R*^2^	0.28		0.20		0.15		0.15	
Degrees of freedom	2543		2543		2543		2543	

* *p* < .05.

** *p* < .01.

*** *p* < .001.

## Highlights

- We examined if multilingualism buffers the negative impact of illiteracy on cognition.
- Illiteracy was associated with worse cognition, while multilingualism had a positive effect.
- Multilingualism did not protect cognition from the negative effect of illiteracy.
- However, multilingualism strengthened the *positive impact of literacy* on cognition.
- Results suggest that multilingualism’s cognitive benefits vary by early-life educational opportunities.

## RESEARCH IN CONTEXT

### Systematic review:

Literature was reviewed using traditional sources (e.g., PubMed). Most studies examining the association of multilingualism on late-life cognition have largely relied on highly educated cohorts from high-income countries. It is unclear whether multilingualism is associated with better cognitive functioning among less privileged groups like illiterate and literate adults with little to no formal schooling such as those residing in India.

### Interpretation:

Our findings suggest that multilingualism contributes differentially to cognition in old age, among individuals with low to no formal schooling, providing benefits for literate adults but limited benefit among illiterate multilingual adults.

### Future directions:

This study provides a strong contribution to understanding the role of multilingualism in late-life cognitive functioning among a linguistically and educationally diverse population. Additional studies are needed to further examine the drivers of multilingual cognitive benefits, including (a) the role of early-life educational opportunities and (b) differences in aspects of multilingualism (i.e., age of acquisition, use, proficiency).

## References

[1] Gallo F, Abutalebi J. The unique role of bilingualism among cognitive reserve-enhancing factors. Biling Lang Cogn. 2023:1–8.

[2] Paap KR, Mason L, Zimiga B, Ayala-Silva Y, Frost M. The alchemy of confirmation bias transmutes expectations into bilingual advantages: a tale of two new meta-analyses. Q J Exp Psychol (Hove). 2020;73(8):1290–1299. 31931663 10.1177/1747021819900098

[3] Paap KR, Johnson HA, Sawi O. Bilingual advantages in executive functioning either do not exist or are restricted to very specific and undetermined circumstances. Cortex. 2015;69:265–278. doi: 10.1016/j.cortex.2015.04.014 26048659

[4] Bak TH. The impact of bilingualism on cognitive ageing and dementia: finding a path through a forest of confounding variables. Linguist Approaches Biling. 2016;6(1–2):205–226.

[5] International Institute for Population Sciences (IIPS), Harvard TH Chan School of Public Health, University of Southern California. Longitudinal ageing study in India (LASI) wave 1, 2017–19, India report Mumbai, India: International Institute for Population Sciences; 2020.

[6] Our World in Data. Healthy life expectancy. Published 2024. Data adapted from: World Health Organization. World Health Statistics 2024 Accessed November 1, 2024. https://ourworldindata.org/healthy-life-expectancy

[7] Nichols E, Steinmetz JD, Vollset SE, Fukutaki K, Chalek J, Abd-Allah F, Estimation of the global prevalence of dementia in 2019 and forecasted prevalence in 2050: an analysis for the Global Burden of Disease Study 2019. Lancet Public Health. 2022;7(2):e105–e125. 34998485 10.1016/S2468-2667(21)00249-8 PMC8810394

[8] Paolillo JC, Das A, Evaluating Language Statistics: The Ethnologue and Beyond. Montreal, Canada: UNESCO Institute for Statistics; 2006. Contract report.

[9] Jolad S, Agarwal A, Mapping India’s language and mother tongue diversity and its exclusion in the Indian Census. Open Science Framework. 2021. doi: 10.31235/osf.io/sjxc6

[10] Office of the Registrar General & Census Commissioner, India. Census of India 2001. New Delhi, India: Office of the Registrar General & Census Commissioner; 2001.

[11] Venkatanarayana M. When will India achieve universal adult literacy?. J Educ Plan Adm. 2015;29(2):177–204.

[12] Nath D. Literacy rate in India 2022. Int J Multidiscip Res. 2023;5(1):1–2.

[13] National Sample Survey Office (NSSO). Survey of Literacy and Educational Attainment. New Delhi, India: Ministry of Statistics and Programme Implementation; 2008.

[14] Arce Rentería M, Vonk JM, Felix G, Avila JF, Zahodne LB, Dalchand E, Illiteracy, dementia risk, and cognitive trajectories among older adults with low education. Neurology. 2019;93(24):e1–e10. doi: 10.1212/WNL.0000000000008587 31722961 PMC6937498

[15] Grosjean F. The bilingual as a competent but specific speaker-hearer. J Multiling Multicult Dev. 1985;6(6):467–477.

[16] Lee J, Khobragade PY, Banerjee J, Design and methodology of the Longitudinal Aging Study in India-Diagnostic Assessment of Dementia (LASI-DAD). J Am Geriatr Soc. 2020;68(S3):S5–S10. doi: 10.1111/jgs.16737 32815602 PMC7503220

[17] Banerjee J, Jain U, Khobragade P, Methodological considerations in designing and implementing the harmonized diagnostic assessment of dementia for longitudinal aging study in India (LASI–DAD). Biodemogr Soc Biol. 2020;65(3):189–213. 10.1080/19485565.2020.1730156 PMC7398273 32727279

[18] Gross AL, Khobragade PY, Meijer E, Saxton JA. Measurement and structure of cognition in the longitudinal aging study in India–diagnostic assessment of dementia. J Am Geriatr Soc. 2020;68(S3):S11–S19. doi: 10.1111/jgs.16738 32815599 PMC7513554

[19] Deaton A. The Analysis of Household Surveys: A Microeconometric Approach to Development Policy. Johns Hopkins University Press; 1997.

[20] Miller GA, Chapman JP. Misunderstanding analysis of covariance. J Abnorm Psychol. 2001;110(1):40–48. 11261398 10.1037//0021-843x.110.1.40

[21] Ljungberg JK, Hansson P, Andrés P, Josefsson M, Nilsson LG. A longitudinal study of memory advantages in bilinguals. PLoS One. 2013;8(9):e73029. 10.1371/journal.pone.0073029 PMC3762844 24023803

[22] Venugopal A, Paplikar A, Varghese FA, Thanissery N, Ballal D, Hoskeri RM, Protective effect of bilingualism on aging, MCI, and dementia: a community-based study. Alzheimers Dement. 2024;20(1):2620–2631. doi: 10.1002/alz.13702 38376105 PMC11032525

[23] Wodniecka Z, Craik FI, Luo L, Bialystok E. Does bilingualism help memory? Competing effects of verbal ability and executive control. Int J Biling Educ Biling. 2010;13(5):575–595.

[24] Paap KR, Greenberg ZI. There is no coherent evidence for a bilingual advantage in executive processing. Cogn Psychol. 2013;66(2):232–258. 23370226 10.1016/j.cogpsych.2012.12.002

[25] Benedikter T, Minority Languages in India: An Appraisal of the Linguistic Rights of Minorities in India. Bozen/Bolzano: EURAC Research Institute for Minority Rights. 2013. Accessed April 28, 2025.

[26] Fuller-Thomson E, Kuh D. The healthy migrant effect may confound the link between bilingualism and delayed onset of Alzheimer’s disease. Cortex. 2014;52:128–130. doi: 10.1016/j.cortex.2013.08.009 24074458

[27] Alladi S, Bak TH, Duggirala V, Surampudi B, Shailaja M, Shukla AK, Bilingualism delays age at onset of dementia, independent of education and immigration status. Neurology. 2013;81(22):1938–1944. 24198291 10.1212/01.wnl.0000436620.33155.a4

[28] Gollan TH, Salmon DP, Montoya RI, Galasko DR. Degree of bilingualism predicts age of diagnosis of Alzheimer’s disease in low-education but not in highly educated Hispanics. Neuropsychologia. 2011;49(14):3826–3830. 22001315 10.1016/j.neuropsychologia.2011.09.041 PMC3223277

[29] Petroysyan S, Strangmann IM, Nichols E, The association of multilingualism with diverse language families and cognition among adults with and without education in India. Neuropsychology;39(3):223–234. 40063370 10.1037/neu0000988 PMC11902890

[30] Valian V. Putting together bilingualism and executive function. Linguist Approaches Biling. 2016;6(5):565–574. doi: 10.1075/lab.15044.val

[31] Manly JJ, Touradji P, Tang MX, Stern Y. Literacy and memory decline among ethnically diverse elders. J Clin Exp Neuropsychol. 2003;25(5):680–690. 12815505 10.1076/jcen.25.5.680.14579

[32] Manly JJ, Schupf N, Tang MX, Stern Y. Cognitive decline and literacy among ethnically diverse elders. J Geriatr Psychiatry Neurol. 2005;18(4):213–217. 16306242 10.1177/0891988705281868

[33] Hernandez SS, Coelho FG, Gobbi S, Stella F. Effects of physical activity on cognitive functions, balance, and risk of falls in elderly patients with Alzheimer’s dementia. Braz J Phys Ther. 2010;14:68–74. 20414564

[34] Wang HX, Jin Y, Hendrie HC, Liang C, Yang L, Cheng Y, Late-life leisure activities and risk of cognitive decline. J GerontolA Biol Sci Med Sci. 2013;68(2):205–213. 10.1093/gerona/gls153 PMC3598354 22879456

[35] van de Vorst IE, Koek HL, Stein CE, Bots ML, Vaartjes I. Socioeconomic disparities and mortality after a diagnosis of dementia: results from a nationwide registry linkage study. Am J Epidemiol. 2016;184(3):219–226. 27380760 10.1093/aje/kwv319

[36] Resende EDPF, Tovar-Moll FF, Ferreira FM, Bramati I, de Souza LC, Carmona KC, White matter microstructure in illiterate and low-literate elderly Brazilians: preliminary findings. Cogn Behav Neurol. 2018;31(4):193–200. 30562228 10.1097/WNN.0000000000000173

[37] Boltzmann M, Mohammadi B, Samii A, Münte TF, Rüsseler J. Structural changes in functionally illiterate adults after intensive training. Neuroscience. 2017;344:229–242. 28069530 10.1016/j.neuroscience.2016.12.049

[38] De Bruin A. Not all bilinguals are the same: a call for more detailed assessments and descriptions of bilingual experiences. Behav Sci (Basel). 2019;9(3):33. 30909639 10.3390/bs9030033 PMC6466537

[39] Mandyla MA, Yannakoulia M, Hadjigeorgiou G, Dardiotis E, Scarmeas N, Kosmidis MH. Identifying appropriate neuropsychological tests for uneducated/illiterate older individuals. J Int Neuropsychol Soc. 2022;28(8):862–875. 34463238 10.1017/S1355617721001016

[40] Jonaitis EM, Koscik RL, Clark LR, Ma Y, Betthauser TJ, Berman SE, Measuring longitudinal cognition: individual tests versus composites. Alzheimers Dement Diagn Assess Dis Monit. 2019;11:74–84. 10.1016/j.dadm.2018.11.006 PMC6816509 31673596

