# Supplementary material for: The association between multilingualism and cognitive function among literate and illiterate older adults with low education in India
Source: Alzheimers Dement Behav Socioecon Aging. Author manuscript; Available in PMC 2026 Jan 20. (PMC12814855; doi:10.1002/bsa3.70018)
Supplement: Supplementary Tables [file NIHMS2112976-supplement-Supplementary_Tables.pdf]

Supplementary Table 1: Demographic characteristics of the matched sample across language and literacy status (*n* = 1,210).

	**Monolingual-Illiterate *n* = 486**	**Multilingual-Illiterate *n* = 484**	**Monolingual-Literate *n* = 119**	**Multilingual-Literate *n* = 121**
**Age,** *M* (*SD*)	70.5 (8.3)	69.7 (7.8)	69.5 (7.3)	70.6 (7.4)
**Sex/gender,** % female	65%	67%	41%	33%
**Years of Education**	0.3 (0.9)	0.4 (1.1)	2.2 (1.8)	2.3 (1.6)
**Parental Education**				
Mother	99% no school	98% no school	90% no school	95% no school
Father	89% no school	86% no school	71% no school	72% no school
**Rurality,** % rural	60%	62%	47%	49%
**Consumption** Median (IQR)	2 (2)	2 (2)	2 (2)	2 (2)

Supplementary Table 2*:* Model estimates per domain in the propensity-matched sample.

	Executive Function		Language		Memory		Visuospatial	
	B	CI	B	CI	B	CI	B	CI
**Illiteracy**	-0.35***	-0.49, -0.22	-0.51***	-0.65, -0.37	-0.28**	-0.45, -0.11	-0.23**	-0.37, -0.08
**Multilingualism**	0.28**	0.12, 0.43	0.20*	0.05, 0.36	0.20*	0.01, 0.39	0.24**	0.08, 0.41
**Illiteracy * Multilingualism**	-0.20*	-0.38, -0.03	-0.15	-0.32, 0.02	-0.22*	-0.43, -0.01	-0.19	-0.37, 0.01
Sex/ gender	-0.26***	-0.33, -0.19	-0.08*	-0.15, -0.01	-0.03	-0.12, 0.06	-0.20***	-0.28, -0.12
Rurality	-0.20***	-0.27, -0.13	-0.09*	-0.16, -0.02	-0.29***	-0.38, -0.20	-0.05	-0.13, 0.02
Age	-0.02***	-0.02, -0.02	-0.02***	-0.02, -0.01	-0.03***	-0.03, -0.02	-0.02***	-0.02, -0.01
Years of Education	0.09***	0.06, 0.12	0.05**	0.02, 0.08	0.07***	0.03, 0.11	0.04**	0.02, 0.08
Mother Education	-0.02	-0.15, 0.10	0.01	-0.12, 0.13	-0.01	-0.16, 0.15	0.03	-0.10, 0.17
Father Education	0.05**	0.02, 0.10	0.03	-0.01, 0.07	0.05*	0.01, 0.10	0.06**	0.02, 0.10
Consumption	0.05**	0.01, 0.08	0.06***	0.02, 0.09	-0.22*	0.03, 0.11	0.02*	-0.02, 0.05
**Adjusted R-squared**	0.32		0.23		0.20		0.15	
**Degrees of freedom**	1,199		1,199		1,199		1,199	

*** p<0.001, ** p<.01, * p<0.05
